# Vitamin D levels in a pediatric population of a primary care centre: a public health problem?

**DOI:** 10.1186/s13104-018-3903-7

**Published:** 2018-11-08

**Authors:** J. M. Fernández Bustillo, A. Fernández Pombo, R. Gómez Bahamonde, E. Sanmartín López, O. Gualillo

**Affiliations:** 1 SERGAS (Servizo Galego de Saude), Pediatric Primary Care, C.S. Bertamiráns, 15220 A Coruña, Bertamirans-Ames, Spain; 2 SERGAS, Division of Endocrinology and Nutrition, University Clinical Hospital of Santiago de Compostela, 15706 Santiago de Compostela, Spain; 3 Musculoskeletal Pathology Group and IDIS (Instituto de Investigación Sanitaria de Santiago), Research Laboratory 18, University Clinical Hospital of Santiago de Compostela, 15706 Santiago de Compostela, Spain; 4 SERGAS (Servizo Galego de Saude) and IDIS (Instituto de Investigación Sanitaria de Santiago), The NEIRID Group (Neuroendocrine Interactions in Rheumatology and Inflammatory Diseases), Research Laboratory 9, University Clinical Hospital of Santiago, Building C, Level-2, Door 9, Travesía da Choupana S/N, Santiago de Compostela, 15706 Spain

**Keywords:** Vitamin D, Vitamin deficiency, Primary care

## Abstract

**Objective:**

Vitamin D deficiency is a public health problem that occurs more frequently than expected. The aim of this study is to evaluate the vitamin D levels of children attending the paediatrics unit of the Bertamiráns primary care centre (A Coruña NW Spain). This is an observational study carried out during 1 year on a random sample of the pediatric population aged between 5 and 15 years. The levels of vitamin D (25(OH)D) were determined by immunoassay (ADVIA Centaur Vitamin D^®^). The results were classified as sufficient (> 20 ng/ml), insufficient (10–20 ng/ml) and deficient (< 10 ng/ml).

**Results:**

153 analyses of vitamin D were carried out (58.2% in girls and 41.8% in boys), distributed in two age groups: 5–10 (62) and 10–15 (91). 66% of the total of the sample presented some degree of vitamin D deficit (60.1% insufficient (92) and 5.9% (11) deficient). In Galicia, there is a high prevalence of vitamin D deficiency/insufficiency in the healthy population, which increases if the patients present some kind of chronic pathology, thus leading to a public health problem. It is advisable to increase the consumption of fortified foods and/or to reconsider the administration of vitamin supplements.

**Electronic supplementary material:**

The online version of this article (10.1186/s13104-018-3903-7) contains supplementary material, which is available to authorized users.

## Introduction

Vitamin D plays a fundamental role in calcium and phosphorus homeostasis and in bone health [1] and its deficit may lead to early consequences regarding mineralization or later complications in the form of osteoporosis. Furthermore, evidence has been shown of its influence on several genes that participate in cell proliferation and differentiation, as well as on the immune system [2]. A lack of vitamin D is one of the most common nutritional realities on a worldwide scale [3] with a high degree of prevalence of vitamin D insufficiency or deficiency in different healthy and unhealthy populations and in pregnant women. Its deficiency has been related to a greater risk of developing many different conditions (autoimmune, infectious, oncological, respiratory, cardiovascular, etc.) and has even been linked to genes related to longevity [4, 5]. Our objective has been to study the levels of 25(OH)D, over a 1-year period, in children undergoing blood testing in the Pediatrics unit of the Bertamiráns primary care centre (A Coruña, NW Spain) for reasons unrelated to the state of their vitamin D levels.

## Main text

This is a descriptive, observational and transversal study. A prospective recruitment of children aged up to 15 years during February 2015 to February 2016 was carried out. Children underwent a standard routine blood test in the Paediatrics unit of the Bertamiráns (Ames, A Coruña) primary care centre, located in north-west Spain (latitude 42°51′36″N, longitude 8°39′0″W). This study was approved by the “Primary Care Directorate” of SERGAS (the Galician regional health service).

All the medical information and analytical data of 153 children (64 boys and 89 girls) was recorded in the electronic medical history of the IANUS software used by the Galician regional health service (SERGAS). The study sample was divided into two age groups: 5–10 years of age (62) and 10–15 years of age (91) with the following data being gathered on a spreadsheet: date of birth; sex; prior illnesses and treatment; vitamin D prophylaxis received, or not, during breastfeeding; analytical and biochemical determinations; calcium phosphorous metabolism; 25(OH)D levels and the date on which the analysis was carried out.

The determination of vitamin D levels was obtained via immunoassay (ADVIA Centaur Vitamin D^®^), in accordance with the manufacturer’s instructions. In accordance with the values obtained, three reference levels were established: sufficiency (> 20 ng/ml), insufficiency (10–20 ng/ml) and deficiency (< 10 ng/ml).

All the subjects in this study were recruited after their parents or legal guardians had signed informed consent forms, excluding those who did not give permission and those who presented some kind of endocrinological pathology linked to bone metabolism. All the procedures have been approved by the SERGAS Ethics Sub-directorate of Primary Health Care and Emergency and by the Sub-directorate of Central Services of the Consellería de Sanidade (the Galician Regional Health Ministry) Edificio Administrativo, San Lázaro, 15703, Santiago de Compostela.

An overall analysis of the results of our research (Table 1 and Additional file 1: Figure S1) shows that only a third of the subjects had sufficient levels of vitamin D (> 20 ng/ml) with the rest (66%) showing low levels. The majority was categorized as insufficient (10–20 ng/ml) whilst 6% proved to be deficient (< 10 ng/ml). The biochemical parameters of the calcium and phosphorus metabolism of all of the subjects were within normal limits.

**Table 1:** Vitamin D levels (ng/ml) ± SEM according to time of year

Season	Analysis (n = 153)	Insufficiency n = 92 (60.01%)	Deficiency n = 9 (5.9%)	Sufficiency n = 52 (33.98%)
Spring	n = 29	n = 20 (68.9%)	n = 3 (10.3%)	n = 6 (20.6%)
		14.91 ± 0.97 ng/ml		23.83 ± 1.01 ng/ml
Summer	n = 37	n = 13 (35.1%)	n = 1 (2.7%)	n = 23 (62.2%)
		17.07 ± 0.81 ng/ml		24.96 ± 0.94 ng/ml
Autumn	n = 34	n = 23 (67.6%)	n = 2 (5.8%)	n = 9 (26.4%)
		15.64 ± 0.77 ng/ml		23.56 ± 0.88 ng/ml
Winter	n = 53	n = 36 (67.9%)	n = 3 (5.6%)	n = 14 (26.4%)
		14.28 ± 0.51 ng/ml		24.86 ± 0.98 ng/ml

A breakdown of the results obtained in relation to the time or season of the year in which the analyses were carried out (Table 1) shows that the period between autumn and spring is when the lowest levels of vitamin D are observed, with this deficiency being more noticeable in the spring (79.3%) compared to 37.8% of insufficiency in the summer period. Notably, when a comparison within the groups (normal and insufficiency) was made along the seasons, a significant difference between vitamin D levels in summer and winter was observed only within the “insufficiency” group, not in the normal group (Additional file 2: Figure S2).

We then went on to form two differentiated groups according to the underlying pathology (Table 2) and, out of the total of the subjects, we were able to observe that 39.21% were suffering from some kind of associated chronic illness (respiratory, overweight/obesity, etc.) with the rest (60.78%) not suffering from any kind of chronic pathology. In this way, we noticed that, in the case of healthy children, the percentage of sufficiency was similar to that of insufficiency/deficiency, although the same did not occur in patients suffering from a pre-existing chronic pathology (overweight/obesity and/or respiratory illness), 80% of whom presented low levels (insufficiency/deficiency) of vitamin D.

**Table 2:** Vitamin D levels (ng/ml) ± SEM in healthy children and those with pathology

Analysis: n = 153	Healthy: n = 93 (60.78%)	Pathology: n = 60 (39.21%)
Sufficiency	n = 40 (43.01%)	n = 12 (20%)
	24.68 ± 0.61 ng/ml	24.33 ± 1.02 ng/ml
Insufficiency/deficiency	n = 53 (56.9%)	n = 48 (80%)
	15.4 ± 0.45 ng/ml	14.19 ± 0.62 ng/ml

The contingency table was analysed with the Fisher’s exact test (p = 0.0049) revealing that the vitamin D levels are related to the state of health of the patients

The group of patients with chronic pathologies can be divided into three subgroups according to their underlying pathology: respiratory, overweight/obesity and a third subgroup including various metabolic (endocrinological) pathologies (not related to bone metabolism), etc. (Table 3).

**Table 3:** Vitamin D levels (ng/ml) ± SEM according to underlying pathology

Pathology, n = 60 (100%)	Respiratory, n = 22 (36.66%)	Overweight/obesity, n = 23 (38.33%)	Others, n = 15 (25%)
Sufficiency, n = 12 (20%)	n = 3 (13.64%)	n = 6 (26.08%)	n = 3 (20%)
	28.3 ± 2.28 ng/ml	22.1 ± 0.65 ng/ml	24.6 ± 2.03 ng/ml
Insufficiency/deficiency, n = 48 (80%)	n = 19 (86.36%)	n = 17 (73.9%)	n = 12 (80%)
	11.6 ± 1.19 ng/ml	14.81 ± 0.62 ng/ml	17.1 ± 0.57 ng/ml

The contingency table was analysed with the Chi square test (p = 0.58) revealing that the vitamin D levels are not related to the underlying pathology of the patients

Considering that 80–90% of vitamin D comes from cutaneous synthesis in mammals [6] originating from the activity of UVB radiation [7], its deficiency is generally related to physical agents which block exposure to solar radiation (skin pigmentation, sun filters, etc.) and/or geographic variables [8]. In this regard, in the northern hemisphere, from around 37°–40° of latitude, the inclination of solar rays leads to a reduction of 80–100% in ultraviolet irradiation during the winter months. All of these factors would lead to an inefficient synthesis, which would explain the seasonal variations in the organic content of vitamin D [9, 10]. This reduction may be accentuated by the use of protective sun creams, which can reduce the cutaneous synthesis of vitamin D by up to 90–95%.

An analysis of the data of our study (Table 1) reveals that the analytical data of vitamin D levels are correlated with the seasonal variation, with the optimum levels of vitamin D being highest in the summer months. However, from the beginning of autumn up to the peak moment in springtime, its insufficiency/deficiency is evident, in line with other studies carried out in neighbouring countries in the European Union [11, 12].

The global prevalence of vitamin D deficit in the group aged between 5 and 10 is significant (56.4%) and reaches 68% in the group aged between 10 and 15 years of age, in line with previously published data, which varies depending on the study in question [13–15].

It must be taken into account that food composition tables state the average vitamin content over the course of the year, without reflecting the seasonal variation of this vitamin. Fish, for example, contains a greater quantity of fat at the end of the summer and, therefore, more vitamin D, with its reserves diminishing over the winter [16]. Indeed, the vitamin D content in fish varies greatly, up to 25–30%, even within the same species, depending on whether it is caught in the wild or bred in captivity (fish farming). Furthermore, the method of cooking can also diminish the vitamin content noticeably [17].

Given that the endogenous production of vitamin D is difficult to quantify, certain daily recommended amounts have been established for different population groups, including a nutritional requirement of 400 IU/day during the first year of life and then 600 IU/day until adolescence [18, 19]. However, in Spain, the real intake of calcium and vitamin D in recent years (from 2001 onwards) has reduced [20] due to the low consumption of food sources with high vitamin D content. Indeed, it can be observed that 85.4% of schoolchildren aged between 7 and 16 have a lower than recommended intake [21] with seasonal variations in vitamin levels and a high percentage, especially in adolescent girls, not reaching the minimum recommended intake of this nutrient [22].

Currently, although no consensus exists regarding recommended serum levels of 25(OH)D, and the cut-off point is variable depending on the criterion employed, the majority of scientific societies consider that deposits are sufficient if their plasma concentration remains above 30 ng/ml [23] and deficient if they are below 20 ng/ml. However, the US Institute of Medicine (IOM) suggests that concentrations of 20 ng/ml are sufficient to protect 97.5% of the population from the harmful effects of vitamin D insufficiency [24]. This point of view is supported by the main pediatric societies (AAP, ESPGHAN, etc.) and the Committee on Nutrition of the AEP (the Spanish Association of Pediatrics) in a recent publication endorsing these recommendations [19].

Based on our data and taking concentrations of 25(OH)D higher than 20 ng/ml as a point of reference, it can be observed that 66% of the subjects presented low levels of vitamin D (Table 1), with this being more evident in the months of autumn and winter, increasing in spring and rising to 80% if there is an underlying chronic pathology (Table 2). However, if the optimum level of vitamin D were established at 30 ng/ml, only 3% of the subjects of the study would have sufficient levels, with the rest (97%) presenting low levels and 67% being classified as having a degree of deficiency (< 10 ng/ml).

It has been suggested that vitamin D, due to its anti-inflammatory and immuno-modulating effect, could have a favourable effect on respiratory infections [25]. This benefit, added to its potential anti-microbial action, could also act to protect against asthma [26], which, added to a possible synergy with corticoids [27], could favour the response to treatment for asthmatic patients. Furthermore, it has been suggested that vitamin D levels could constitute a potentially modifiable marker of asthma [28], associating the deficit of 25(OH)D to an increase in the prevalence of this condition on a worldwide scale.

Likewise, as it is a fat-soluble vitamin, vitamin D deficit has also been related to obesity due to the fact that it is stored in fatty tissue, of which these patients have a large volume and more supply is needed to fill the deposits [29]. Its deficit, therefore, is associated to diabetes mellitus (types 1 and 2) due to beta cell dysfunction, with a reduction of secretion and an increase of insulin resistance [30].

In our case study, 40% of the subjects (Table 2) presented an associated underlying pathology (respiratory, metabolic, obesity, etc.). In these cases, the levels of vitamin D insufficiency/deficiency were higher than those of the healthy population, affecting 80% of the subjects. We proceeded to classify them into three subgroups (Table 3) according to the predominant pathology. It was observed that, of those with some kind of respiratory pathology (allergy, asthma), 86% had low levels of 25(OH)D, which reduced to 74% in overweight/obese subjects, in line with the findings of other studies [25, 26]. The third subgroup included children with diverse pathologies (endocrinological, metabolic, inflammatory bowel disease, malabsorption, etc.). However, the degree of insufficiency/deficiency also affected 80% of the subjects analysed.

In conclusion, taking as a reference point the recommendations of ESPGHAN and IOM, which define values of ≥ 20 ng/dl as sufficient, we have detected a high prevalence of vitamin D deficiency/insufficiency in the pediatric population participating in our study, particularly in the months of winter and spring. During these periods, 79% of the subjects did not achieve optimum levels, with this figure extending to 97% if the value of sufficiency were established at ≥ 30 ng/ml, as recommended by the Endocrine Society. Therefore, we recommend a scientific consensus in order to establish the optimum level of vitamin D and, given the difficulty of maintaining an organic content of this vitamin throughout the year in the pediatric population, advise an evaluation of the necessity of consuming larger quantities of natural dietary sources or foods rich in vitamin D and/or reconsidering the current recommendations in favour of the administration of vitamin supplements [29, 30].

## Limitations

One possible limitation of this study, in addition to the fact that it is transversal, could be the age of the children making up the sample (5–15 years of age) as well as their number, as it may not be sufficiently representative in order to detect deficiency or to allow values lower than the average to be discriminated. Furthermore, the lack of a nutritional survey of the sample being studied should be taken into account, along with time exposed to the sun, as there is the possibility that the true situation is not adequately reflected.

## Additional files

**Additional file 1.** Distribution of vitamin D levels across the seasons of the year in the studied population. In all the seasons the differences are highly significant between the studied groups (normal levels vs insufficiency). Data have been analysed with a non-parametric test (Mann–Whitney test). *** p < 0.0001.

**Additional file 2.** Comparison between vitamin D levels along the seasons within the groups (normal and insufficiency). In the insufficiency group the differences between summer and winter vitamin D levels are statistically significant. However, the normal group did not show any significant variation in vitamin D levels along the seasons. Data have been analysed with a non-parametric test (Kruskal–Wallis test) followed by a Dunns multiple comparison test. * p < 0.05.

## Abbreviations

AAP: American Academy of Pediatrics
AEP: Asociación Española de Pediatría
ESPGHAN: European Society for Paediatric Gastroenterology Hepatology and Nutrition
IOM: Institute of Medicine
SERGAS: Servizo Galego de Saúde
