# Supplementary material for: Vitamin D levels in a pediatric population of a primary care centre: a public health problem?
Source: BMC Res Notes. 2018 Nov 8;11:801. doi: 10.1186/s13104-018-3903-7 (PMC6225586; doi:10.1186/s13104-018-3903-7)
Supplement: Supplementary file 1 — Additional file 1. Distribution of vitamin D levels across the seasons of the year in the studied population. In all the seasons the differences are highly significant between the studied groups (normal levels vs insufficiency). Data have been analysed with a non-parametric test (Mann–Whitney test). *** p < 0.0001. [file 13104_2018_3903_MOESM1_ESM.pptx]

## Slide 1
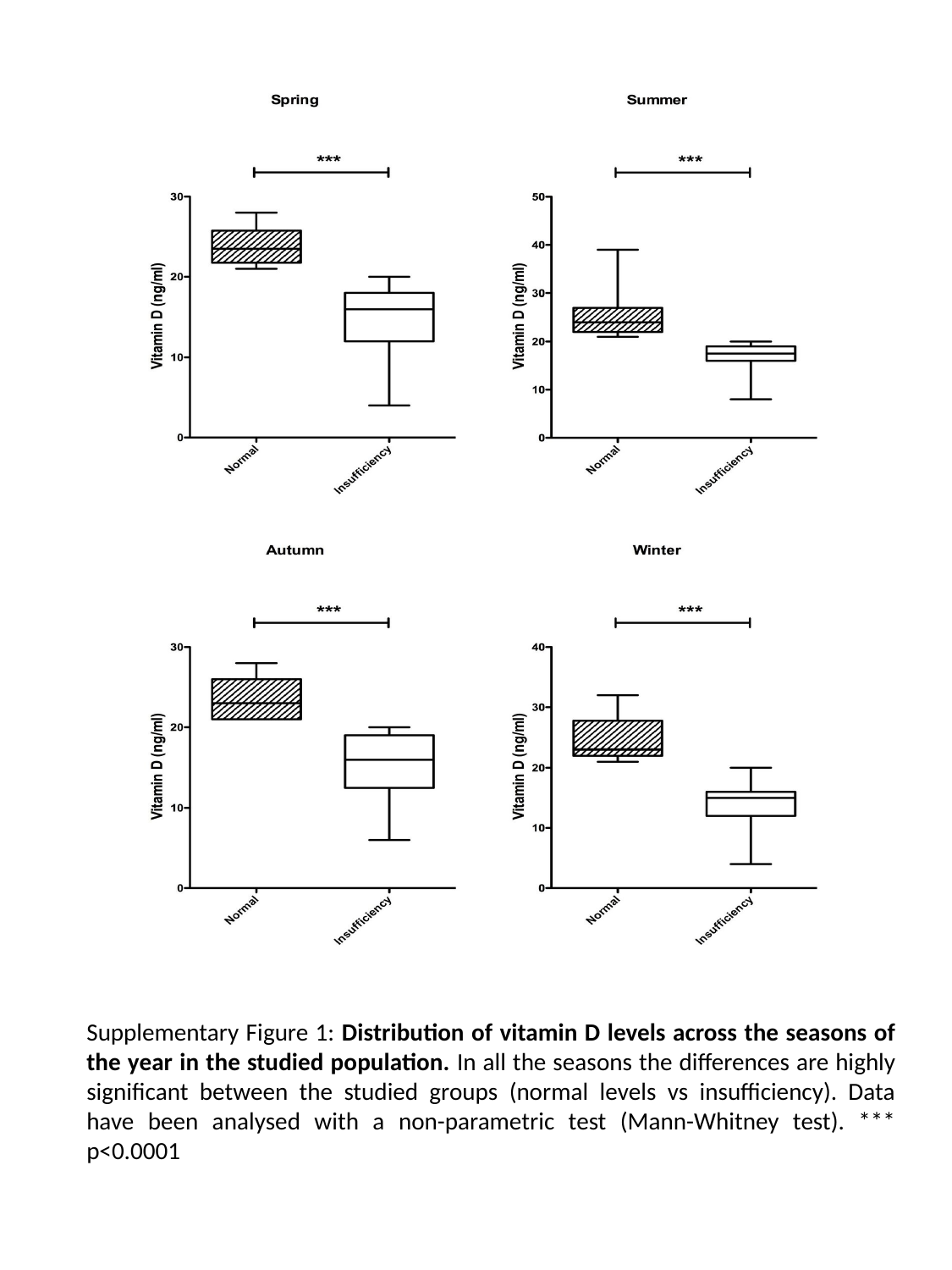

Supplementary Figure 1: Distribution of vitamin D levels across the seasons of the year in the studied population. In all the seasons the differences are highly significant between the studied groups (normal levels vs insufficiency). Data have been analysed with a non-parametric test (Mann-Whitney test). *** p<0.0001
