# Supplementary material for: Vitamin D levels in a pediatric population of a primary care centre: a public health problem?
Source: BMC Res Notes. 2018 Nov 8;11:801. doi: 10.1186/s13104-018-3903-7 (PMC6225586; doi:10.1186/s13104-018-3903-7)
Supplement: Supplementary file 2 — Additional file 2. Comparison between vitamin D levels along the seasons within the groups (normal and insufficiency). In the insufficiency group the differences between summer and winter vitamin D levels are statistically significant. However, the normal group did not show any significant variation in vitamin D levels along the seasons. Data have been analysed with a non-parametric test (Kruskal–Wallis test) followed by a Dunns multiple comparison test. * p < 0.05. [file 13104_2018_3903_MOESM2_ESM.pptx]

## Slide 1
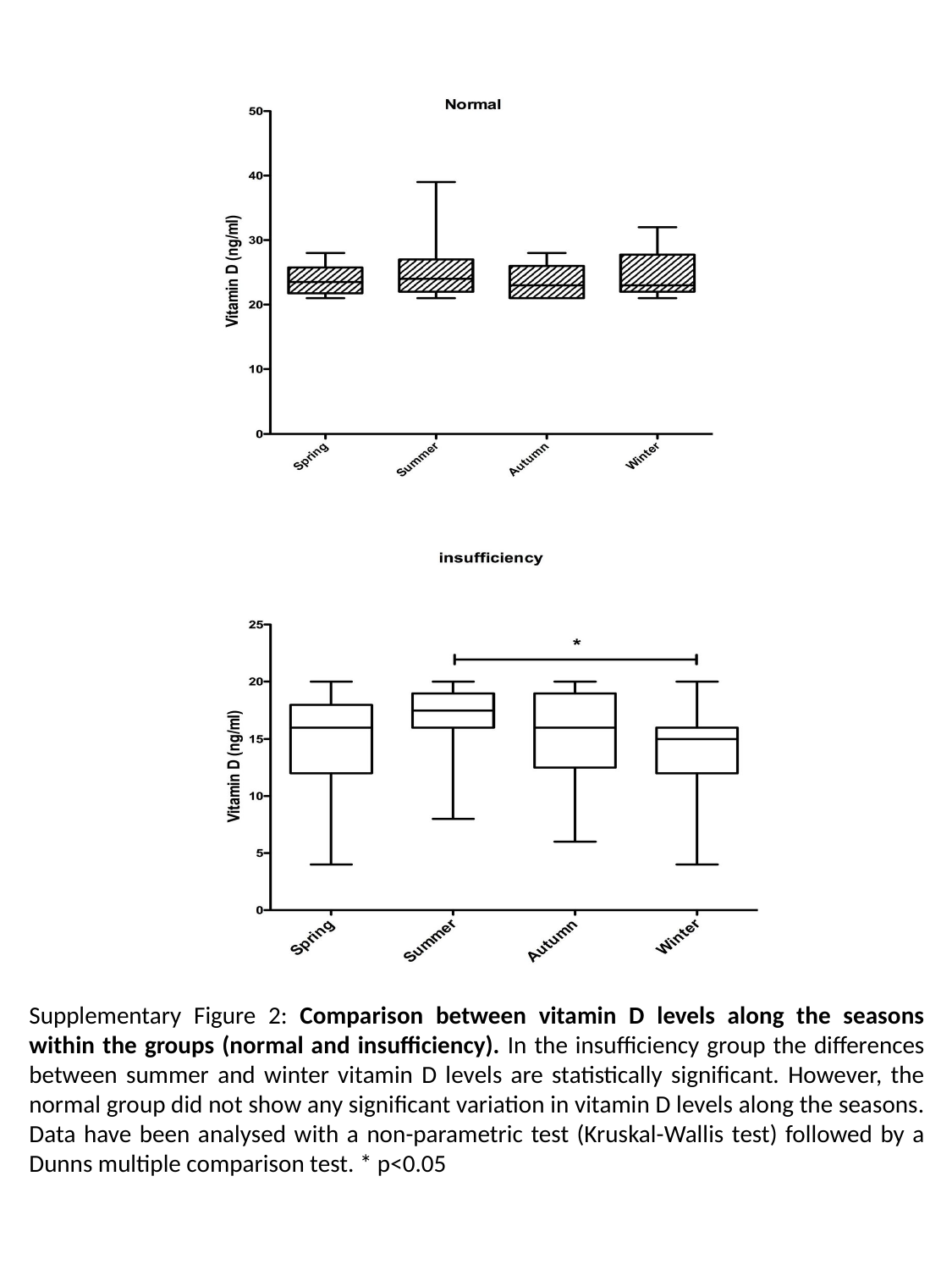

Supplementary Figure 2: Comparison between vitamin D levels along the seasons within the groups (normal and insufficiency). In the insufficiency group the differences between summer and winter vitamin D levels are statistically significant. However, the normal group did not show any significant variation in vitamin D levels along the seasons. Data have been analysed with a non-parametric test (Kruskal-Wallis test) followed by a Dunns multiple comparison test. * p<0.05
